# Exploring the causal relationship between inflammatory bowel disease and sarcopenia-related traits: a two-sample Mendelian randomization analysis

**DOI:** 10.18632/aging.205421

**Published:** 2023-12-31

**Authors:** Shangjin Lin, Chaobao Zhang, Cong Chen, Yongqian Fan, Fengjian Yang

**Affiliations:** 1 Department of Orthopedics, Huadong Hospital Affiliated to Fudan University, Shanghai 200040, China; 2 Shanghai Key Laboratory of Clinical Geriatric Medicine, Shanghai 200040, China

**Keywords:** Mendelian randomization, inflammatory bowel disease, sarcopenia, appendicular lean mass, hand grip strength

## Abstract

Previous observational studies have shown an association between inflammatory bowel disease (IBD) and sarcopenia. However, the causal relationship between IBD (including ulcerative colitis and Crohn’s disease) and sarcopenia remains unclear. Thus, this study investigated whether genetically predicted IBD play a function in the occurrence of sarcopenia using Mendelian randomization (MR) analysis. This study used independent single nucleotide polymorphisms (SNPs) significantly associated with IBD as instrument variables (IVs). Sarcopenia-related components (hand grip strength, walking space, and appendicular lean mass (ALM)) were investigated as outcome factors, with summary-level data regarding these components of sarcopenia obtained from the UK Biobank. The IVW-MR analysis revealed that there were significant negative associations between IBD and hand grip strength (both left and right) as well as ALM. Besides, the results of IVW-MR analysis provided strong evidence of a causal relationship between genetically predicted Crohn’s disease and hand grip strength and ALM. However, there were no significant associations found between ulcerative colitis and sarcopenia-related traits. Sensitivity tests confirmed the accuracy and robustness of the above MR analysis. Conclusions: Our MR analysis showed the causal effect of Crohn’s disease on hand grip strength and ALM. This suggests that Crohn’s disease may be a potential risk factor for sarcopenia.

## INTRODUCTION

Human life expectancy has continuously been extended through advances in society and medical technology, leading to an increasingly prominent trend of population aging. Global aging has led to an increase in chronic diseases related to aging, which has been a significant burden on health care and medical insurance funds. Sarcopenia is a complex multifactorial disease strongly associated with aging, characterized by loss of skeletal muscle mass, and weakening of muscle strength [1]. Among European adults over 80 years, sarcopenia prevalence rates range from 15 to 39% among men and 19 to 50% among women [2]. An imbalance between protein synthesis and catabolism in skeletal muscle cells primarily causes sarcopenia. Sarcopenia is known to be closely related to aging, chronic inflammation, oxidative stress, malnutrition, and genetic factors [3–6]. However, the etiology of sarcopenia remains unknown.

Inflammatory bowel disease (IBD) is a group of chronic and recurrent intestinal diseases, including ulcerative colitis (UC) and Crohn’s disease (CD). UC mainly involves the colon and rarely involves the small intestine [7]. In UC, inflammation usually begins in the rectum and can spread continuously to involve either a portion or the entirety of the colon [8]. Unlike Crohn’s disease, UC primarily affects the inner lining of the bowel wall and typically does not extend beyond the colon [9]. Conversely, the CD can affect any part of the digestive tract from the mouth to the anus, but it most commonly affects the terminal ileum and colon [10]. Inflammation in CD is usually patchy and affects the entire thickness of the bowel wall, which can result in complications like strictures and fistulas [11]. Research on the potential relationship between IBD and sarcopenia has recently garnered substantial attention from numerous scholars. The relationship between these two conditions is complex, with multiple potential mechanisms contributing to muscle deterioration in patients with IBD. One significant factor contributing to sarcopenia in individuals with IBD is malnutrition. Malnutrition is often observed in IBD patients due to factors like reduced nutrient absorption and increased nutritional needs [12]. Additionally, chronic inflammation, a characteristic of IBD, plays a crucial role in the development of sarcopenia [3]. This persistent state of inflammation can result in heightened catabolic stress on skeletal muscle tissues, further worsening muscle wasting. The latest research data found that the prevalence of sarcopenia in patients with IBD was 52% and 37%, respectively [13]. A retrospective study found that sarcopenia was an independent predictor of surgical complications in IBD patients younger than 40 years [14]. Furthermore, a recent cross-sectional study demonstrated that 41.3% of 344 patients with IBD in clinical remission were diagnosed with sarcopenia and possible sarcopenia [15]. Importantly, sarcopenia in IBD has been linked to adverse outcomes, suggesting that it is a critical aspect of the disease that warrants attention and further research. Numerous observational studies have found a correlation between IBD and sarcopenia, but it is unclear whether there is a causal link between them. Therefore, in this study, we performed a two-sample Mendelian randomization (MR) analysis to clarify the causal relationship between IBD (including UC and CD) and sarcopenia.

MR is a scientific technique that uses genetic mutations or single nucleotide polymorphisms (SNPs) as biomarkers to evaluate causal relationships between exposures and outcomes in a natural setting [16]. This method relies on information on genetic variation to serve as an instrumental variable for surrogate exposure, allowing the assessment of the causal effect between exposure factors and outcome variables. A MR analysis of the correlation between IBD and sarcopenia has not been documented in the literature yet. No randomized controlled trial (RCT) has yet been conducted to assess the causal relationship between these two variables. Therefore, a two-sample MR analysis was conducted to investigate the causal relationship between IBD (including UC and CD) and sarcopenia (measured by hand grip strength, appendicular lean mass, and walking pace) in this research.

## RESULTS

### Filtering of instrumental variables

Our study aimed to examine the causal relationship between IBD (including UC and CD) and sarcopenia. To achieve this, we first obtained 117, 87 and 122 linkage disequilibrium (LD)-independent (R^2^ < 0.001) SNPs significantly associated with IBD, UC and CD (*p* < 5 × 10–8) as instrumental variables (IVs) for exposure factors. Supplementary Tables 1–3 provided detailed information on the IVs for IBD, UC, and CD. From the outcome genome-wide association study (GWAS) summary data, the SNPs for the selected IVs were extracted. The selected SNPs would be removed in the following situations: firstly, during the process of extracting specific SNPs from the GWAS related with sarcopenia, the requested SNPs were not present and the proxies that were in LD with the requested SNPs could not be identified in the outcome GWAS. Secondly, palindromic SNPs with intermediate allele frequencies were removed after harmonizing the effect allele. Finally, after implementing the Mendelian randomization pleiotropy residual sum and outlier (MR-PRESSO) method to eliminate pleiotropic outlier SNPs, the number of SNPs chosen as IVs for exposure in subsequent analyses would be the same as or fewer than those listed in Supplementary Tables 1–3. In this study, the F-statistics corresponding to each IV were significantly greater than 10, indicating a minimal likelihood of weak instrumental variable bias.

### Causality of genetically predicted IBD (including UC and CD) on hand grip strength

**Figure 1:**
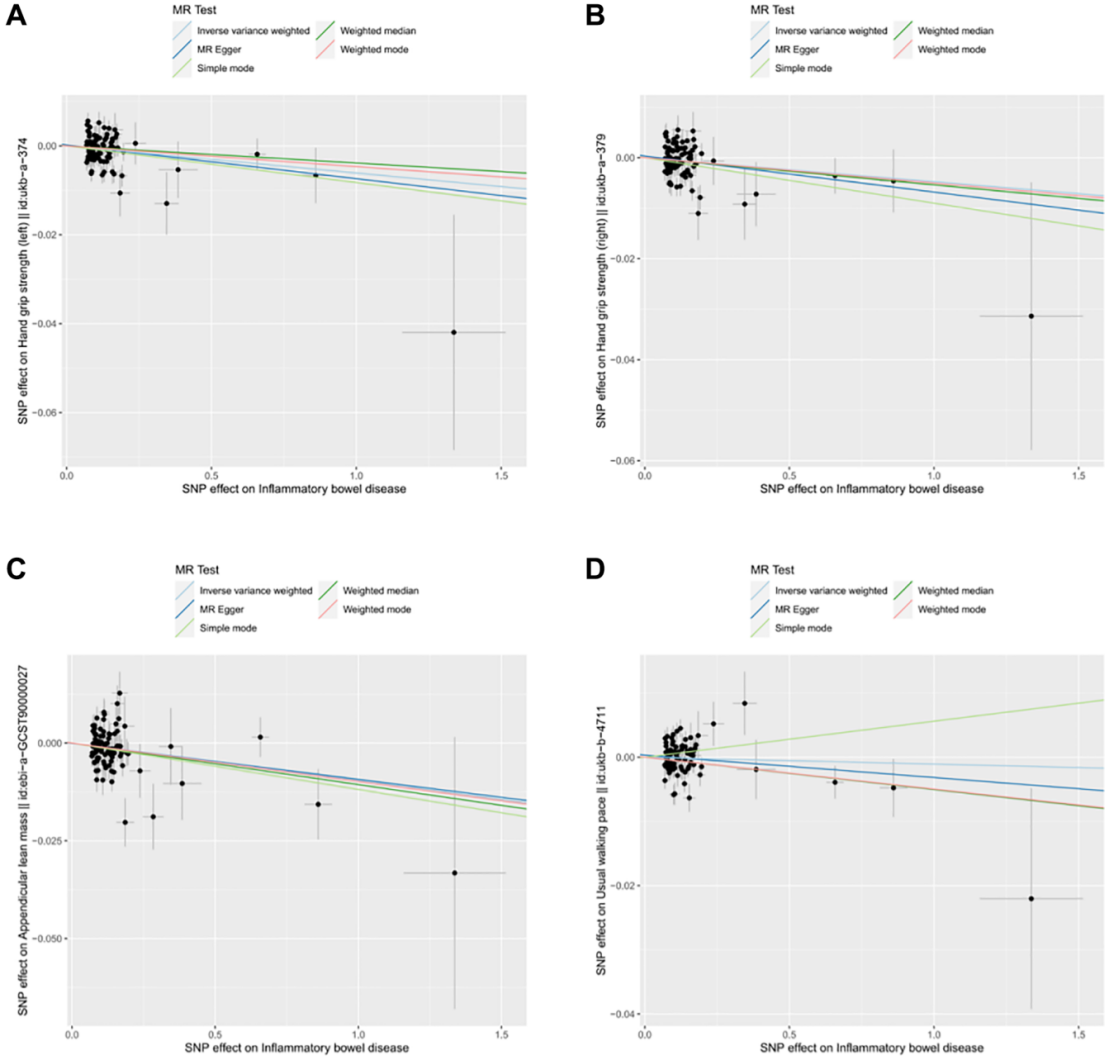
**Scatter plot for MR analyses of the causal effect of IBD on sarcopenia after removing the pleiotropic outlier SNPs.** (**A**) IBD-grip strength (left). (**B**) IBD-grip strength (right). (**C**) IBD-ALM. (**D**) IBD-Walking pace.

**Figure 2:**
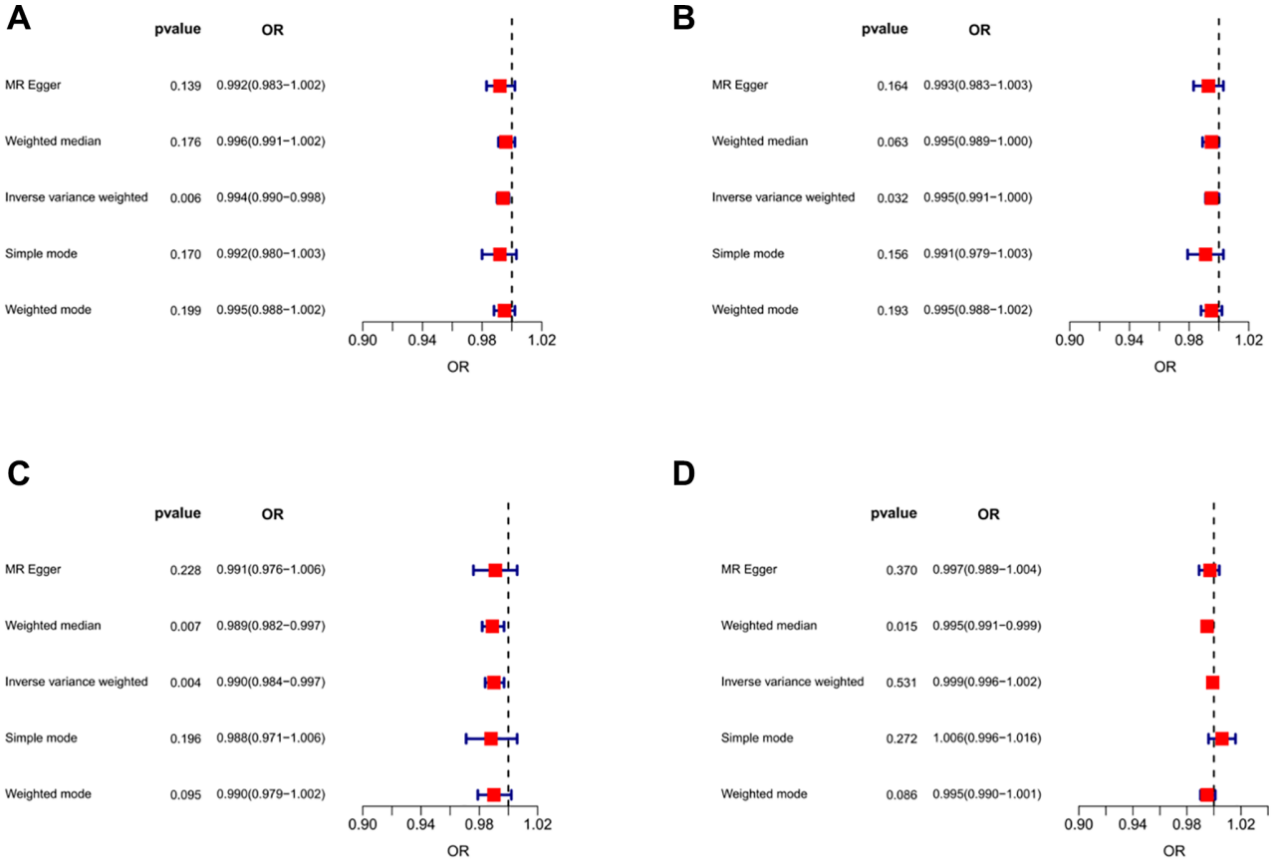
**Forest plots of MR results for assessing the causal effect of IBD on sarcopenia.** (**A**) IBD-grip strength (left). (**B**) IBD-grip strength (right). (**C**) IBD -ALM. (**D**) IBD -Walking pace.

**Figure 3:**
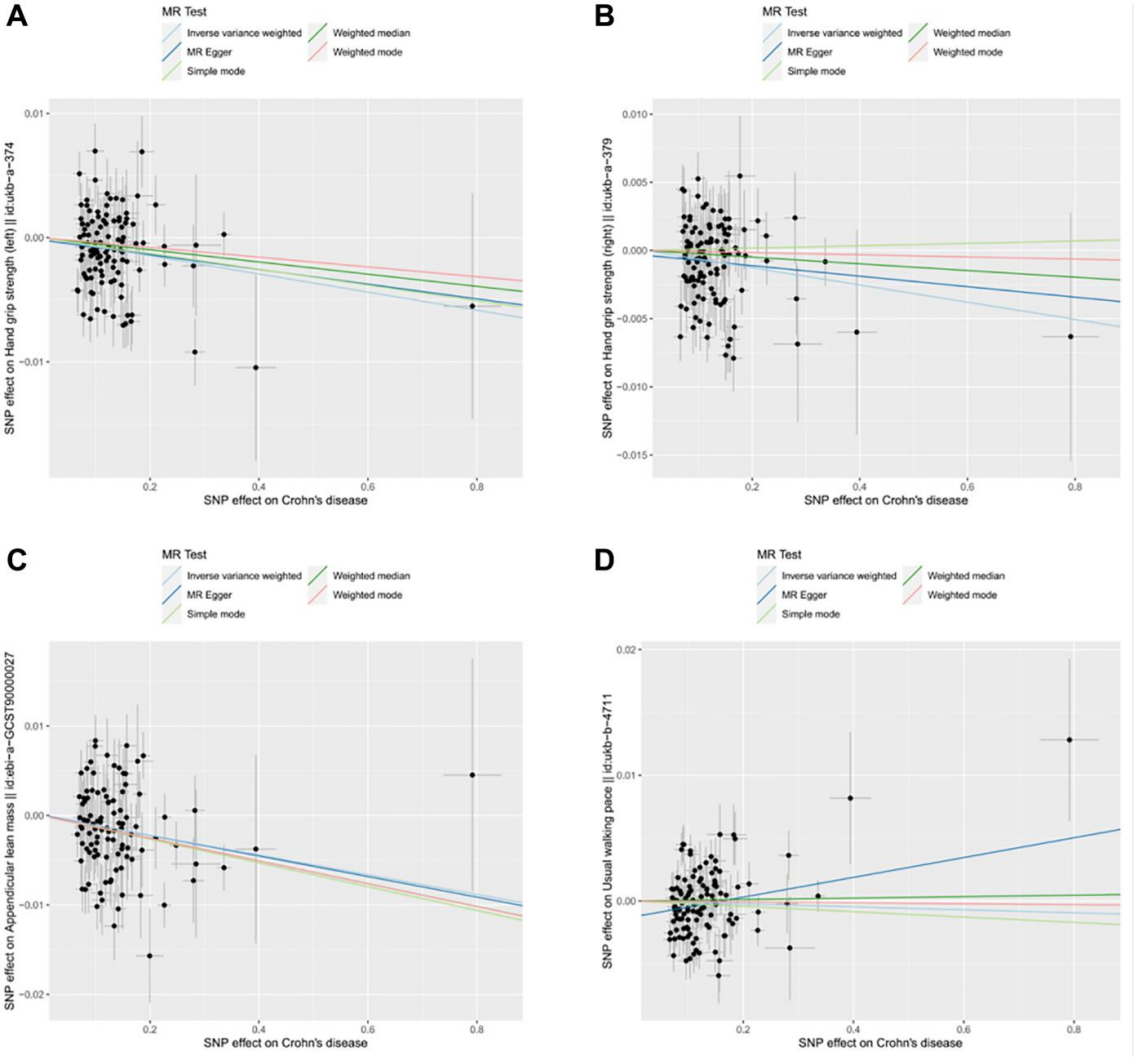
**Scatter plot for MR analyses of the causal effect of CD on sarcopenia after removing the pleiotropic outlier SNPs.** (**A**) CD-grip strength (left). (**B**) CD-grip strength (right). (**C**) CD-ALM. (**D**) CD-Walking pace.

**Figure 4:**
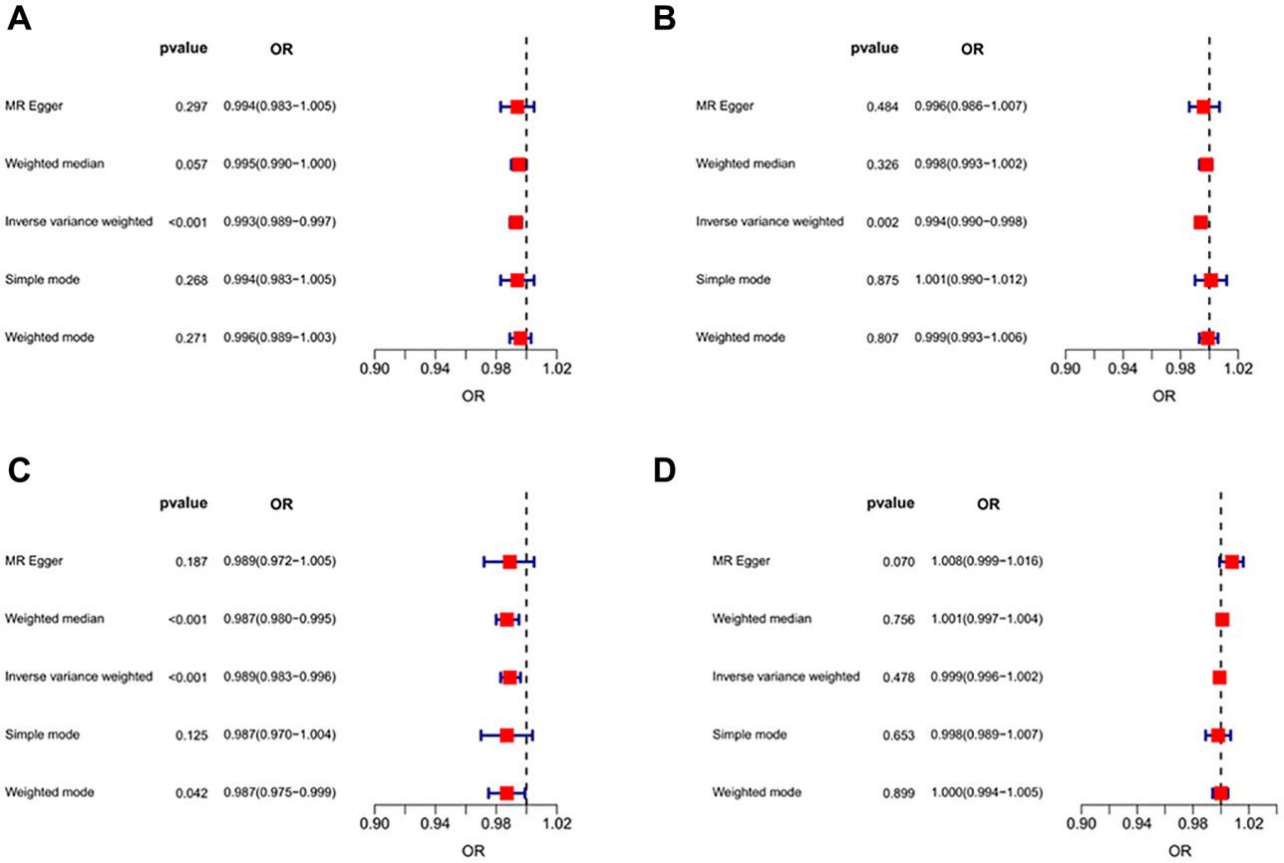
**Forest plots of MR results for assessing the causal effect of CD on sarcopenia.** (**A**) CD-grip strength (left). (**B**) CD-grip strength (right). (**C**) CD-ALM. (**D**) CD-Walking pace.

**Figure 5:**
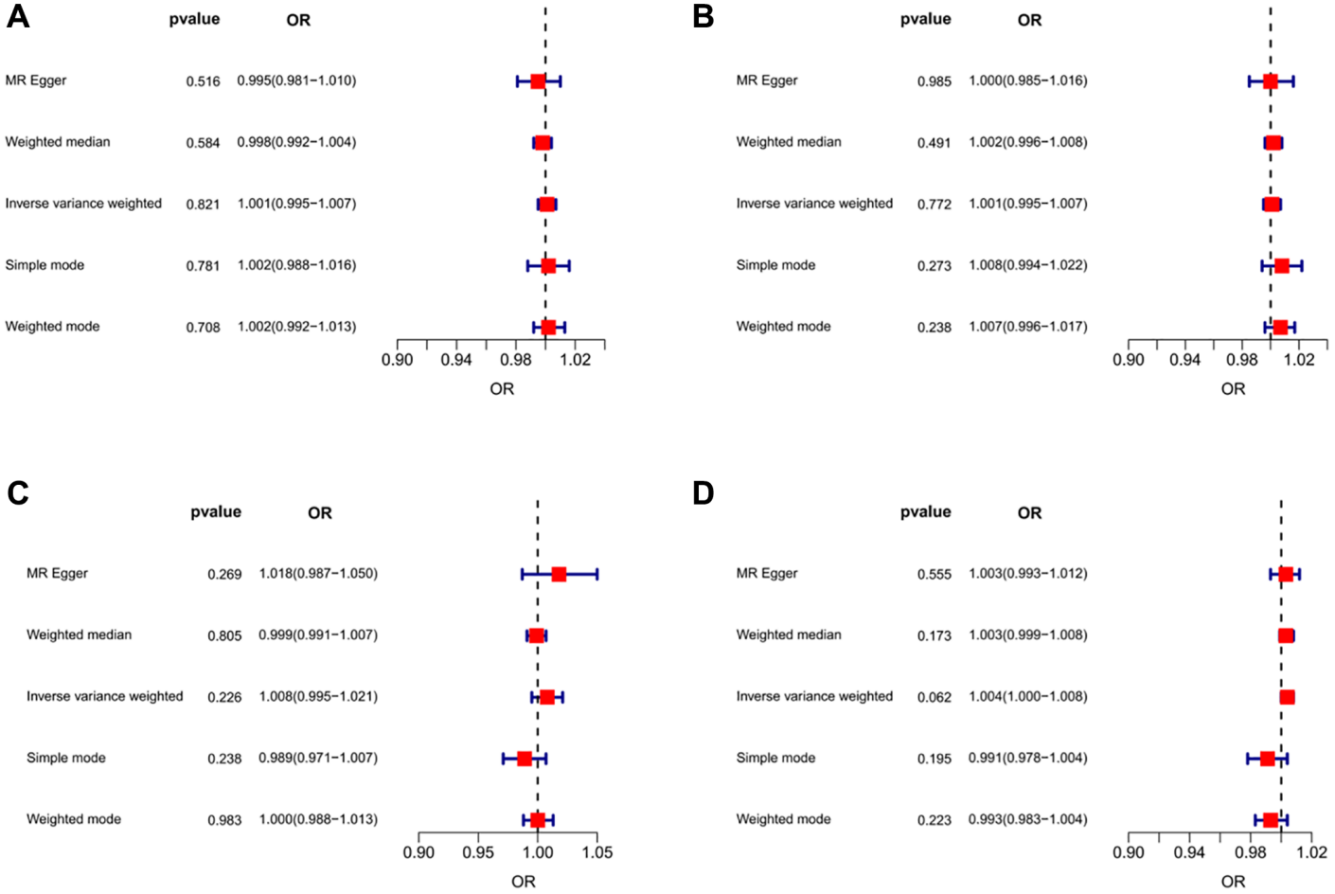
**Forest plots of MR results for assessing the causal effect of UC on sarcopenia.** (**A**) UC-grip strength (left). (**B**) UC-grip strength (right). (**C**) UC-ALM. (**D**) UC-Walking pace.

**Figure 6:**
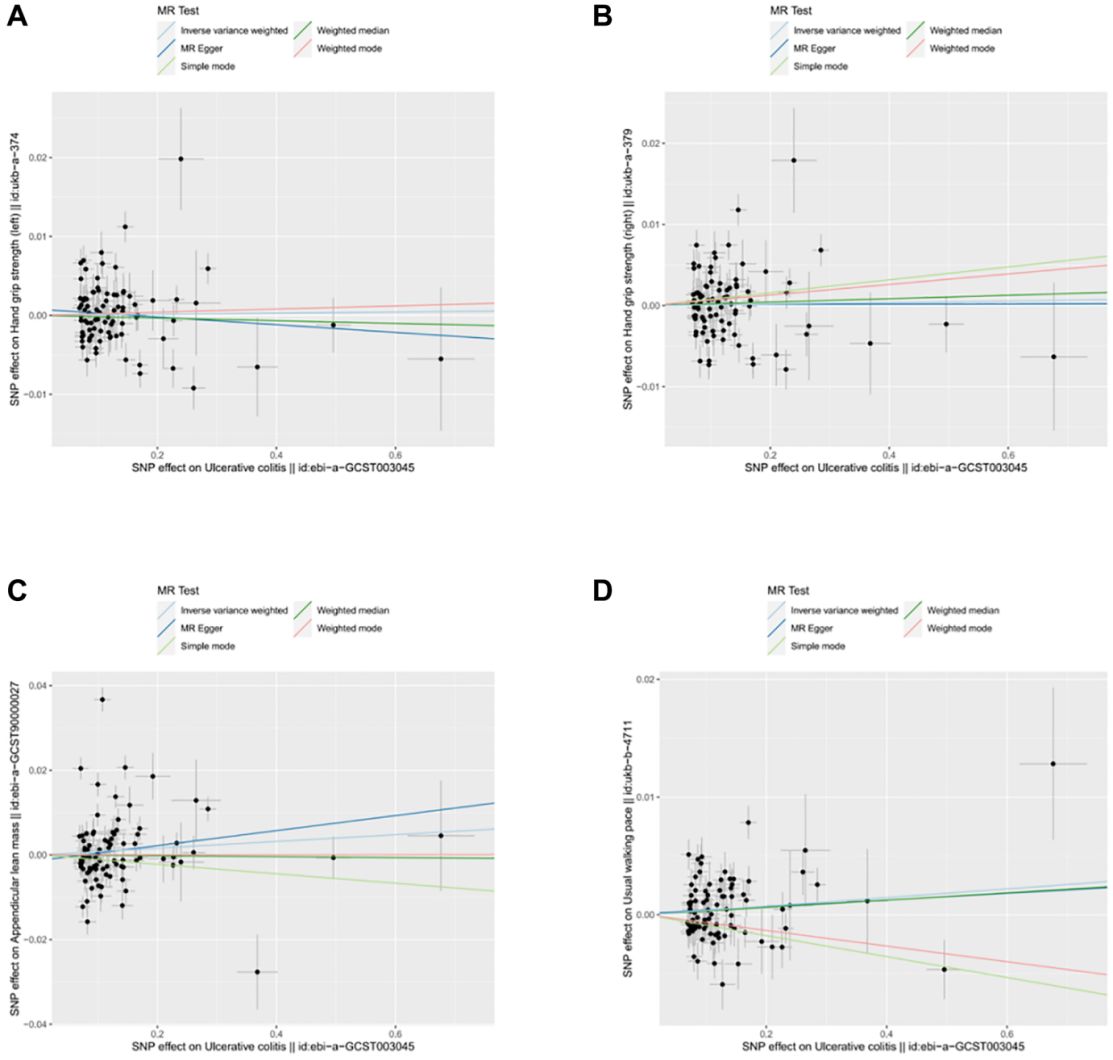
**Scatter plot for MR analyses of the causal effect of UC on sarcopenia after removing the pleiotropic outlier SNPs.** (**A**) UC-grip strength (left). (**B**) UC-grip strength (right). (**C**) UC-ALM. (**D**) UC-Walking pace.

The results of inverse variance weighted (IVW)-MR method (see Figures 1A, 1B and 2A, 2B) revealed a potential causal effect of IBD on hand grip strength (Left: OR = 0.994, 95% CI = 0.990–0.998, *p* = 0.006; Right: OR = 0.995, 95% CI = 0.991–1.000, *p* = 0.032), with detail in Table 1. Similar outcomes (see Figures 3A, 3B and 4A, 4B) were obtained from the results in the causal effect of CD on hand grip strength (Left: OR = 0.993, 95% CI = 0.989–0.997, *p <* 0.001; Right: OR = 0.994, 95% CI = 0.990–0.998, *p* = 0.002), with detail in Table 2. In contrast, no significant causal relationship was found between UC and hand grip strength (Left: OR = 1.001, 95% CI = 0.995–1.007, *p* = 0.821, Figure 5A; Right: OR = 1.001, 95% CI = 0.995–1.007, *p* = 0.772, Figure 5B), as detailed in Table 3 and Figures 5, 6. The MR Egger regression analysis did not show any evidence of directional pleiotropy between SNPs associated with IBD (including UC and CD). Based on the heterogeneity test results (*p* < 0.001), IVW-MR was used in the random-effects model. The plots of the leave-one-out method analysis indicate that the results of the MR analysis remained stable and were not influenced by any individual SNP (Supplementary Figures 1–3).

**Table 1:** The MR results from different methods for assessing the causal effect of IBD on sarcopenia.

**Outcomes**	**No. of IVs**	**Methods**	**Beta**	**SE**	**OR**	**95% CI (OR)**	***P*-value**
Hand grip strength (left)	95	MR Egger	−0.008	0.005	0.992	0.983–1.002	0.139
	95	Weighted median	−0.004	0.003	0.996	0.991–1.002	0.176
	95	IVW	−0.006	0.002	0.994	0.990–0.998	**0.006**
	95	Simple mode	−0.008	0.006	0.992	0.980–1.003	0.170
	95	Weighted mode	−0.005	0.004	0.995	0.988–1.002	0.199
Hand grip strength (right)	95	MR Egger	−0.007	0.005	0.993	0.983–1.003	0.164
	95	Weighted median	−0.005	0.003	0.995	0.989–1.000	0.063
	95	IVW	−0.004	0.002	0.995	0.991–1.000	**0.032**
	95	Simple mode	−0.009	0.006	0.991	0.979–1.003	0.156
	95	Weighted mode	−0.005	0.004	0.995	0.988–1.002	0.193
Appendicular lean mass	95	MR Egger	−0.009	0.008	0.991	0.976–1.006	0.228
	95	Weighted median	−0.011	0.004	0.989	0.982–0.997	**0.007**
	95	IVW	−0.010	0.003	0.990	0.984–0.997	**0.004**
	95	Simple mode	−0.012	0.009	0.988	0.971–1.006	0.196
	95	Weighted mode	−0.010	0.006	0.990	0.979–1.002	0.095
Usual walking pace	93	MR Egger	−0.003	0.004	0.997	0.989–1.004	0.370
	93	Weighted median	−0.005	0.002	0.995	0.991–0.999	**0.015**
	93	IVW	−0.001	0.002	0.999	0.996–1.002	0.531
	93	Simple mode	0.006	0.005	1.006	0.996–1.016	0.272
	93	Weighted mode	−0.005	0.003	0.995	0.990–1.001	0.086

**Table 2:** The MR results from different methods for assessing the causal effect of CD on sarcopenia.

**Outcomes**	**No. of IVs**	**Methods**	**Beta**	**SE**	**OR**	**95% CI (OR)**	***P*-value**
Hand grip strength (left)	113	MR Egger	−0.006	0.006	0.994	0.983–1.005	0.297
	113	Weighted median	−0.005	0.003	0.995	0.990–1.000	0.057
	113	IVW	−0.007	0.002	0.993	0.989–0.997	**<0.001**
	113	Simple mode	−0.006	0.006	0.994	0.983–1.005	0.268
	113	Weighted mode	−0.004	0.004	0.996	0.989–1.003	0.271
Hand grip strength (right)	113	MR Egger	−0.004	0.005	0.996	0.986–1.007	0.484
	113	Weighted median	−0.002	0.002	0.998	0.993–1.002	0.326
	113	IVW	−0.006	0.002	0.994	0.990–0.998	**0.002**
	113	Simple mode	0.001	0.006	1.001	0.990–1.012	0.875
	113	Weighted mode	−0.001	0.003	0.999	0.993–1.006	0.807
Appendicular lean mass	102	MR Egger	−0.011	0.009	0.989	0.972–1.005	0.187
	102	Weighted median	−0.013	0.004	0.987	0.980–0.995	**<0.001**
	102	IVW	−0.011	0.003	0.989	0.983–0.996	**<0.001**
	102	Simple mode	−0.013	0.009	0.987	0.970–1.004	0.125
	102	Weighted mode	−0.013	0.006	0.987	0.975–0.999	**0.042**
Usual walking pace	113	MR Egger	0.008	0.004	1.008	0.999–1.016	0.070
	113	Weighted median	0.001	0.002	1.001	0.997–1.004	0.756
	113	IVW	−0.001	0.002	0.999	0.996–1.002	0.478
	113	Simple mode	0.002	0.005	0.998	0.989–1.007	0.653
	113	Weighted mode	−0.001	0.003	1.000	0.994–1.005	0.899

**Table 3:** The MR results from different methods for assessing the causal effect of UC on sarcopenia.

**Outcomes**	**No. of IVs**	**Methods**	**Beta**	**SE**	**OR**	**95% CI (OR)**	***P*-value**
Hand grip strength (left)	84	MR Egger	−0.005	0.007	0.995	0.981–1.010	0.516
	84	Weighted median	−0.002	0.003	0.998	0.992–1.004	0.584
	84	IVW	0.001	0.003	1.001	0.995–1.007	0.821
	84	Simple mode	0.002	0.007	1.002	0.988–1.016	0.781
	84	Weighted mode	0.002	0.005	1.002	0.992–1.013	0.708
Hand grip strength (right)	84	MR Egger	0.001	0.008	1.000	0.985–1.016	0.985
	84	Weighted median	0.002	0.003	1.002	0.996–1.008	0.491
	84	IVW	0.001	0.003	1.001	0.995–1.007	0.772
	84	Simple mode	0.007	0.007	1.008	0.994–1.022	0.273
	84	Weighted mode	0.006	0.005	1.007	0.996–1.017	0.238
Appendicular lean mass	85	MR Egger	0.018	0.016	1.018	0.987–1.050	0.269
	85	Weighted median	−0.001	0.004	0.999	0.991–1.007	0.805
	85	IVW	0.008	0.007	1.008	0.995–1.021	0.226
	85	Simple mode	−0.011	0.009	0.989	0.971–1.007	0.238
	85	Weighted mode	0.001	0.006	1.000	0.988–1.013	0.983
Usual walking pace	84	MR Egger	0.003	0.005	1.003	0.993–1.012	0.555
	84	Weighted median	0.003	0.002	1.003	0.999–1.008	0.173
	84	IVW	0.004	0.002	1.004	1.000–1.008	0.062
	84	Simple mode	−0.009	0.007	0.991	0.978–1.004	0.195
	84	Weighted mode	−0.007	0.005	0.993	0.983–1.004	0.223

### Causality of genetically predicted IBD (including UC and CD) on ALM

After applying strict exclusion criteria, 95, 102, and 85 SNPs were identified for IBD, CD, and UC in our MR analysis. All these SNPs met the criteria for having an F-statistic >10, indicating the absence of weak instrumental variable bias. Tables 1–3 show the analysis results of the five methods for the MR analysis. Given our large number of IVs, Cochran’s *Q* test showed heterogeneity (*p* < 0.05). Therefore, we chose the random effects model in the IVW-MR analysis. There was a significant negative causal relationship between IBD and ALM (OR = 0.990, 95% CI = 0.984–0.997, *p* = 0.004; Figures 1C and 2C, Table 1). Similarly, the IVW analysis showed that CD had a negative causal effect on ALM (OR = 0.989, 95% CI = 0.983–0.996, *p <* 0.001; Figures 3C and 4C, Table 2). Besides, the results obtained from the weighted median method (IBD: OR = 0.989, 95% CI = 0.982–0.997, *p* = 0.007; CD: OR = 0.987, 95% CI = 0.980–0.995, *p <* 0.001) were in agreement with those of the IVW method. However, no causal effect of UC on ALM was found in this section (OR = 1.008, 95% CI = 0.995–1.021, *p* = 0.226; Figure 5C, Table 3). Based on the MR-Egger regression results, there was no evidence of pleiotropy among IVs in this section. The absence of horizontal pleiotropy for the SNPs included in this MR analysis was also confirmed. The absence of horizontal pleiotropy for the SNPs included in this MR analysis was also confirmed through visual inspection of the funnel plots, as shown in Supplementary Figures 4–6.

### Causality of genetically predicted IBD (including UC and CD) on walking pace

Our study identified 93, 113, and 84 SNPs as IVs for IBD, CD, and UC when the walking pace was used as an outcome factor. Similarly, we used the random effects model in IVW-MR analysis due to the heterogeneity among SNPs. Unfortunately, our IVW-MR analysis did not reveal a significant causal relationship between IBD and walking pace (OR=0.999, 95% CI = 0.996–1.002, *p* = 0.531; Figures 1D and 2D, Table 1). Although the weighted median provided clear evidence of a causal association between IBD and walking pace in the MR analysis (OR = 0.995, 95% CI = 0.991–0.999, *p* = 0.015), we refrained from finding a causal relationship between the two variables due to the dominance of the IVW technique. As shown in Table 2, we also did not detect a causal effect of CD on walking pace (Figures 3D and 4D). Likewise, the various MR methods employed did not reveal a significant causal relationship between UC and walking speed, with specific details provided in Table 3 and Figure 5D.

## DISCUSSION

The European Working Group on Sarcopenia in Older People (EWGOP) defines sarcopenia as a syndrome characterized by a decrease in muscle mass, strength, and function due to various factors that affect the body’s physiological and metabolic functions [2]. IBD is a chronic gastrointestinal disease that causes chronic inflammation and ulceration of the intestinal mucosa, leading to intestinal bleeding, diarrhea, abdominal pain, and other symptoms. The cause of IBD is not yet fully understood, but skeletal muscle impairment is a common pathological feature of this disease [17]. Furthermore, numerous studies have shown that the inflammatory state of the intestinal tract in patients with IBD may serve as a trigger for the onset of muscle loss by activating several cellular signaling pathways that are commonly associated with sarcopenia [18–20]. By exploring the causal link between IBD and sarcopenia, we can offer novel perspectives on the development and lay the groundwork for potential treatment strategies aimed at regulating the gut-muscle axis.

Although previous cross-sectional studies have shown a correlation between IBD and sarcopenia, the causal relationship between these two pathological conditions has yet to be explored. This study used the two-sample MR method and large-scale GWAS data to investigate the potential causal link between IBD (including UC and CD) and the three components of traits (hand grip strength, ALM, and walking pace). The results of our MR analysis demonstrated that IBD and CD had significant negative association with hand grip strength and ALM. Genetic evidence, however, did not support the association between UC and sarcopenia-related traits. The variation in the MR results for causal association between CD and UC may be explained by the fact that CD involves systemic involvement and widespread inflammation in the gastrointestinal tract, whereas UC is characterized by more localized inflammation. The presence of transmural inflammation and the possibility of small bowel involvement in CD may contribute to more pronounced malabsorption problems, potentially worsening malnutrition and skeletal muscle wasting [21]. Conversely, the inflammation in UC, although still severe, generally does not affect the small intestine, which is the primary site for nutrient absorption, possibly resulting in a different sarcopenia profile compared to CD. Consequently, these findings have clinical significance as they highlight the importance of proactive sarcopenia management in CD to reduce negative health outcomes. However, this link seems to be less prominent in UC patients.

The hand grip strength test is a widely used non-invasive method for evaluating upper-limb muscle strength and overall physical fitness [22]. It is a simple test that provides valuable information about an individual’s physical condition. The research findings showed that there was a significant negative causal effect between CD and hand grip strength, which is supported by genetic evidence. Previous observational studies have found a negative correlation between CD and hand grip strength, corroborating our findings. A cross-sectional study was conducted on 150 patients with CD and 254 controls, which revealed that patients with CD had significantly lower hand grip strength and hand grip strength index compared to the controls (*p* < 0.0001) [23]. This study suggests that the hand grip strength index can serve as a reliable and valuable parameter to predict functional nutritional status and skeletal muscle health in patients with CD.

ALM, which refers to lean muscle mass and strength, is a term used in body composition analysis to describe the amount of lean tissue in the limbs of the human body. It is an important indicator of general skeletal muscle in individuals. The assessment of ALM can be done using various methods, such as dual X-ray absorptiometry (DXA), bioelectric impedance analysis (BIA), and magnetic resonance imaging (MRI). These methods allow accurate muscle mass measurement and are used in clinical settings to assess patients at risk for muscle wasting and sarcopenia. Previous studies have found that CD is closely related to ALM. An observational study found that the skeletal muscle mass index in CD patients was significantly lower than in controls (6.0 +/− 1.1 versus 6.5 +/− 1.2; *p* < 0.05) [24]. Another research on 137 pre-menopausal CD patients aged 18 to 50 gathered cross-sectional data and discovered that low lean body mass and sarcopenia were prevalent among CD patients [25]. However, most of these studies were cross-sectional observational studies, and their conclusions only suggested a correlation between CD and ALM. Our MR analysis found that CD was causally negatively correlated with ALM (OR = 0.989, 95% CI = 0.983–0.996, *p <* 0.001), which provided a higher level of evidence to support previous research results.

A slow walking pace can indicate reduced muscle strength and function [26]. Walking requires the coordination of multiple muscle groups, including the legs and core, and is based on muscle strength and power to propel the body forward. As muscle mass and strength decline with age, gait speed, and walking ability may be affected, leading to a slower pace. A case-control study observed that the gait speed of 23 women with UC was slower compared to the control group of 23 healthy women (−17%, *p* = 0.002) [27]. However, our MR analysis did not find a causal effect between IBD (including UC and CD) and walking pace. The GWAS data of the walking pace utilized in this study were acquired from the UKB. It is important to note that most participants in the UKB were relatively young and healthy, which may impact the results.

The study represents the first two-sample MR analysis to investigate the relationship between IBD (including UC and CD) and three sarcopenia-associated traits. The findings of this study are significant as they provide novel insights into the potential causal relationship between CD and sarcopenia. MR analysis has advantages over previous observational research in addressing bias and inferring causality. MR analysis uses genetic variants as IVs, which are less susceptible to confounding or reverse causality compared to environmental or lifestyle factors measured in observational studies. This helps to minimize the potential for bias in the estimates of the causal effects of the exposure on the outcome. This study used an iterative approach to confirm the consistency of the estimates both before and after removing outliers, ultimately strengthening the evidence presented. Moreover, sensitivity analyses were performed to ensure the consistency of causal forecasts and confirm the findings’ robustness. Overall, these measures contribute to the reliability and validity of the conclusions of these MR analyses.

Nonetheless, there are some limitations to this research. Due to inadequate comprehension and diagnosis of sarcopenia, the GWAS database failed to discover genetic variation data linked to sarcopenia. Nevertheless, as hand grip strength, walking space, and ALM are reliable predictors of sarcopenia, we assert that this study provides substantial evidence of a negative causal relationship between CD and sarcopenia. Besides, our study utilized ALM data from the UKB, which was measured using BIA. It is important to note that BIA is an indirect measurement method and may be less accurate than DXA, which directly quantifies the mass of the skeletal muscle. Finally, the GWAS data used in this research were limited to individuals of European ancestry. Therefore, further research to determine whether the conclusions of our study can be applied to other ethnic groups. In conclusion, this study offers valuable insights into the intricate relationship between IBD and sarcopenia. The results indicate a strong causal link between genetically predicted CD and sarcopenia, emphasizing CD as a potential risk factor for sarcopenia. This connection emphasizes the significance of proactive management of sarcopenia in patients with CD to reduce the risk of skeletal muscle wasting and related complications.

## MATERIALS AND METHODS

### Study design

In this study, we conducted a two-sample MR analysis to investigate the causal relationship between IBD (including UC and CD) and sarcopenia-related traits. The summary-level data for our MR analysis were obtained from GWAS databases. We conducted a search in GWAS databases to identify genetic variants associated with IBD, CD, and UC as exposure factors. The genetic variants used as IVs needed to meet three core assumptions of MR analysis [28], including the following: (1) hypothesis of significant association between IVs and exposure factors; (2) the IVs were independent of any confounding factors related to the exposure or outcome factors; (3) IVs could only influence the outcome through exposure. Our analysis focused on examining the causal effect of these exposure factors on sarcopenia-related traits. Besides, we conducted pleiotropy and sensitivity analyses to evaluate the accuracy of the MR results. These methods were utilized to address potential biases and confounding factors.

### Data sources for IBD

For a more reliable understanding of the correlation between IBD and sarcopenia, we used GWAS data from a large sample of individuals with UC or CD diagnoses [29]. The above GWAS summary statistics for IBD (including UC and CD), presented in Table 4, are available for download from the IEU GWAS database (https://gwas.mrcieu.ac.uk/datasets/). The included participants were all European of descent to minimize the effect of the population stratification variables on the outcomes.

**Table 4:** Details of the GWAS summary statistics used in the research.

	**Trait**	**Participants**	**Number of SNPs**	**Population**	**Datatype**	**GWAS ID**
**Exposure**	Inflammatory bowel disease	25042 cases and 34915 controls	9619016	European	Binary	ebi-a-GCST004131
	Ulcerative colitis	6968 cases and 20462 controls	110944	European	Binary	ebi-a-GCST003045
	Crohn’s disease	17897 cases and 33977 controls	124888	European	Binary	ieu-a-12
**Outcomes**	Hand grip strength (left)	335,821	10,894,596	European	Continuous	ukb-a-374
	Hand grip strength (right)	335,842	10,894,596	European	Continuous	ukb-a-379
	Appendicular lean mass	244,730	18,164,071	European	Continuous	ebi-a-GCST90000027
	Usual walking pace	459,915	9,851,867	European	Continuous	ukb-b-4711

### Data sources for sarcopenia-related traits

As a common assessment indicator of sarcopenia, the grip strength test is a simple and efficient method of evaluating hand muscle function. The GWAS summary statistics for grip strength were acquired from the United Kingdom Biobank (UKB), including 335,842 individuals for right-grip strength and 335,821 for left-grip strength. Calculating ALM is an effective method to measure muscle mass indirectly and plays an irreplaceable role in diagnosing sarcopenia. ALM is considered a superior method for assessing muscle mass compared to whole-body lean mass. This is because ALM reduces the impact of systemic water, non-adipose soft tissue, cardiac muscle, and vascular smooth muscle on muscle mass [2]. The GWAS summary statistics for ALM were collected from the UKB in a discovery cohort of 244,730 individuals [30]. The GWAS data used BIA to measure ALM and adjusted for potential confounding factors such as fat mass, height, sex, age, and other covariates. Walking pace, closely associated with muscle function and balance, can serve as a fast and secure screening indicator for sarcopenia. Summary statistics for the usual walking pace were obtained from the UKB, with data collected from 459,915 individuals. To minimize the potential impact of population stratification, all participants included above were of European descent. Cohort details regarding exposure and outcomes are presented in Table 4.

### Selection of instrumental variables

Prior to conducting MR analysis, several quality control measures were implemented on the GWAS summary data for IBD (including UC and CD) to identify instrumental SNPs as IVs that met the required criteria. The genome-wide SNPs that showed a significant association with IBD, UC, and CD (*p* < 5 × 10–8) were initially identified to support the first hypothesis of MR analysis. The process of clumping (R^2^ < 0.001, window size = 10,000 kb) was executed on the European samples obtained from the 1000 Genomes Project data to eliminate SNPs that exhibit pairwise LD [31]. Next, the PhenoScanner database (http://www.phenoscanner.medschl.cam.ac.uk/) was utilized to search for information on each IV extracted from the IBD GWAS to determine whether the candidate IVs met the requirements of core assumptions 2 and 3. Thirdly, we searched for the requested IVs from the GWAS summary statistics of sarcopenia-related traits. If a requested IV was not found in the outcome GWAS, we searched for a proxy IV in LD with the requested IVs. For each identified proxy SNP, we extracted the effect alleles, allele frequencies, and associated statistics from the GWAS summary data. Lastly, we harmonized the SNP data with the outcome datasets to ensure consistency in allele coding and to avoid any strand ambiguities. This harmonization step plays a crucial role in aligning the exposure and outcome datasets, thereby minimizing the potential bias in our causal estimates.

### Mendelian randomization analysis

In this study, we conducted a two-sample MR analysis with IBD (including UC and CD) as the exposure factor and sarcopenia-related traits as the outcome factor to explore the potential causal effect of IBD on sarcopenia. Five MR methods were employed to confirm the causal relationship between IBD and sarcopenia, including IVW, Mendelian randomization-Egger, weighted mode, weighted median, and simple mode methods, among which IVW method was the most robust method for MR analysis. The IVW method employs an inverse-variance weighted meta-analysis approach to amalgamate Wald estimates for each IV, resulting in an overall estimate of the effect of IBD on sarcopenia [32]. The selection between the IVW fixed-effects model and the IVW random-effects model was based on the presence or absence of significant heterogeneity among the IVs (*p* < 0.05). Cochran’s *Q* test was used to detect the presence of significant heterogeneity between IVs [33]. By utilizing the weighted mean method, we ensure that the estimate of the causal effect is not unduly influenced by any single variant and accurately represents the average of the distribution of the causal estimates [34]. The leave-one-out test is another robust analytical tool used in the research. It involves systematically excluding each genetic variant from the analysis to evaluate the impact of individual variants on the overall causal estimate. Besides, the funnel plot is a crucial visual tool utilized to identify asymmetry and potential bias in the data. A symmetrical distribution of data points in the funnel plot indicates that the results are not influenced by pleiotropy or other violations of the instrumental variable assumptions. To minimize the influence of genetic variation or measurement errors derived from population data on MR results, we employed MR-PRESSO to detect and eliminate pleiotropic IVs [35]. The MR-PRESSO analysis was utilized for identifying and rectifying outliers in the linear regression of IVW. For this study, the distribution number in the MR-PRESSO analysis was established as 3000. Finally, we performed the MR analysis after deleting pleiotropic outlier IVs using MR-PRESSO. The MR-Egger intercept test is also a valuable tool for detecting potential horizontal pleiotropy among IVs [36]. All the above methods were conducted in R version 4.1.3, and the R packages used included “TwoSampleMR” and “MRPRESSO”. The study used publicly available GWAS summary data, eliminating the need for approval from the Ethics Committee. The results of MR analysis showed the relative risk of IBD-induced sarcopenia by OR and 95% CI. A *p*-value of less than 0.05 was considered statistically significant.

### Data availability statement

The data used in this study were obtained from publicly available datasets, which can be accessed through the MR-Base platform.

## Supplementary Materials

Supplementary Figures

Supplementary Table 1

Supplementary Table 2

Supplementary Table 3
