# Supplementary material for: Exploring the causal relationship between inflammatory bowel disease and sarcopenia-related traits: a two-sample Mendelian randomization analysis
Source: Aging (Albany NY). 2023 Dec 31;16(1):799–819. doi: 10.18632/aging.205421 (PMC10817405; doi:10.18632/aging.205421)
Supplement: Supplementary Figures [file aging-16-205421-s001.pdf]

SUPPLEMENTARY FIGURES

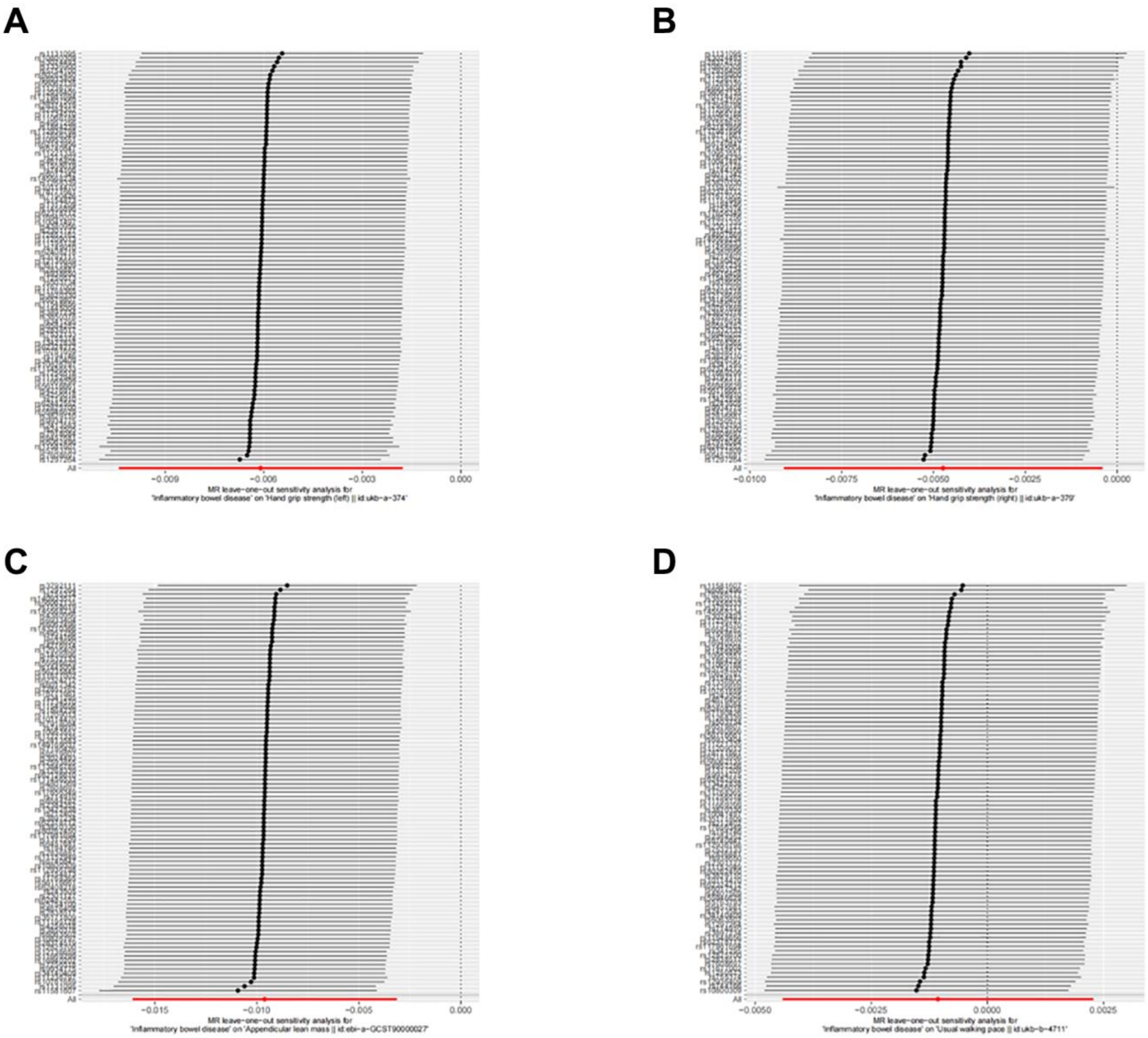

Supplementary Figure 1. Leave-one-out analysis in the MR analyses of the causal effect of IBD on sarcopenia. (**A**) IBD-grip strength (left). (**B**) IBD-grip strength (right). (**C**) IBD-ALM. (**D**) IBD-Walking pace.

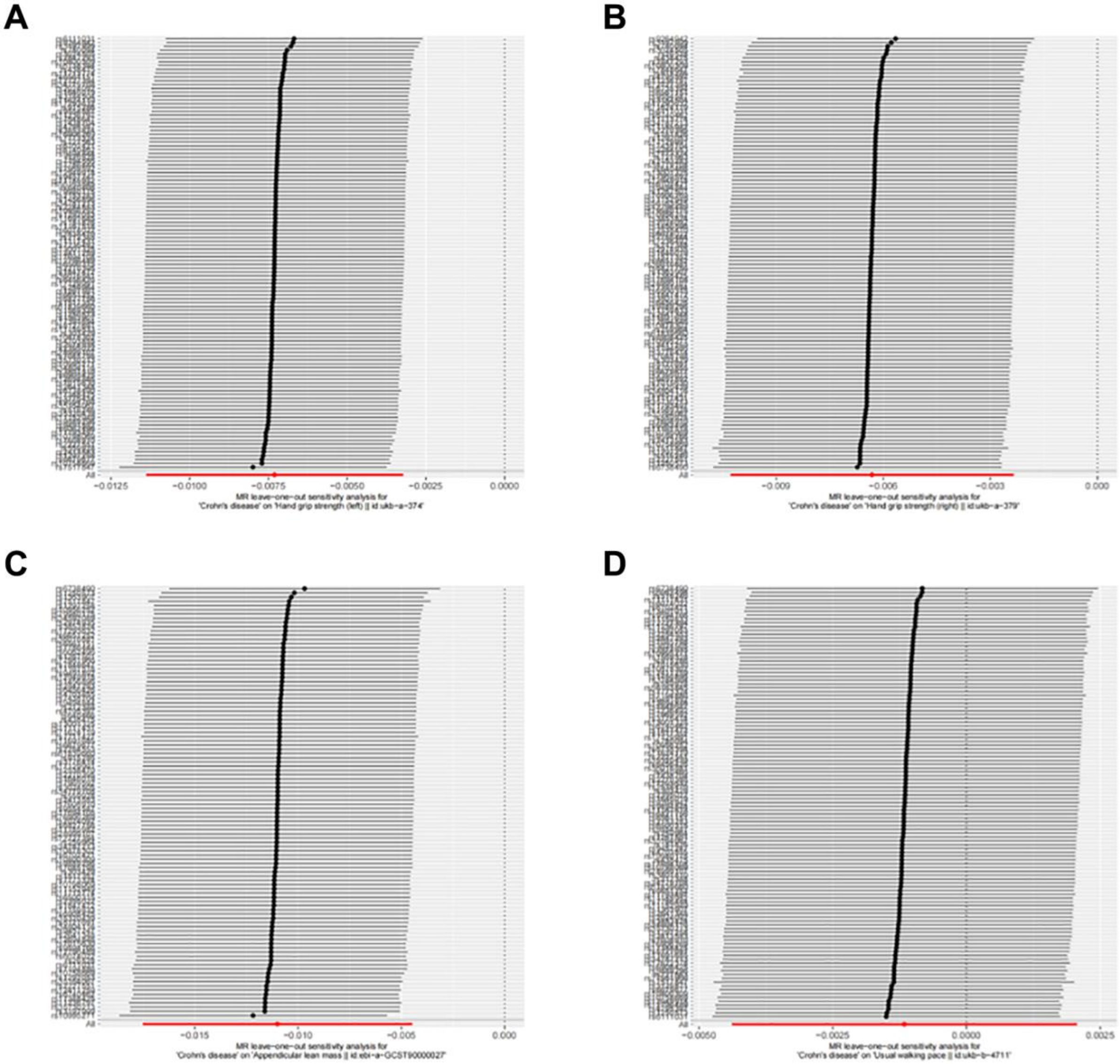

Supplementary Figure 2. Leave-one-out analysis in the MR analyses of the causal effect of CD on sarcopenia. (**A**) CD-grip strength (left). (**B**) CD-grip strength (right). (**C**) CD-ALM. (**D**) CD-Walking pace.

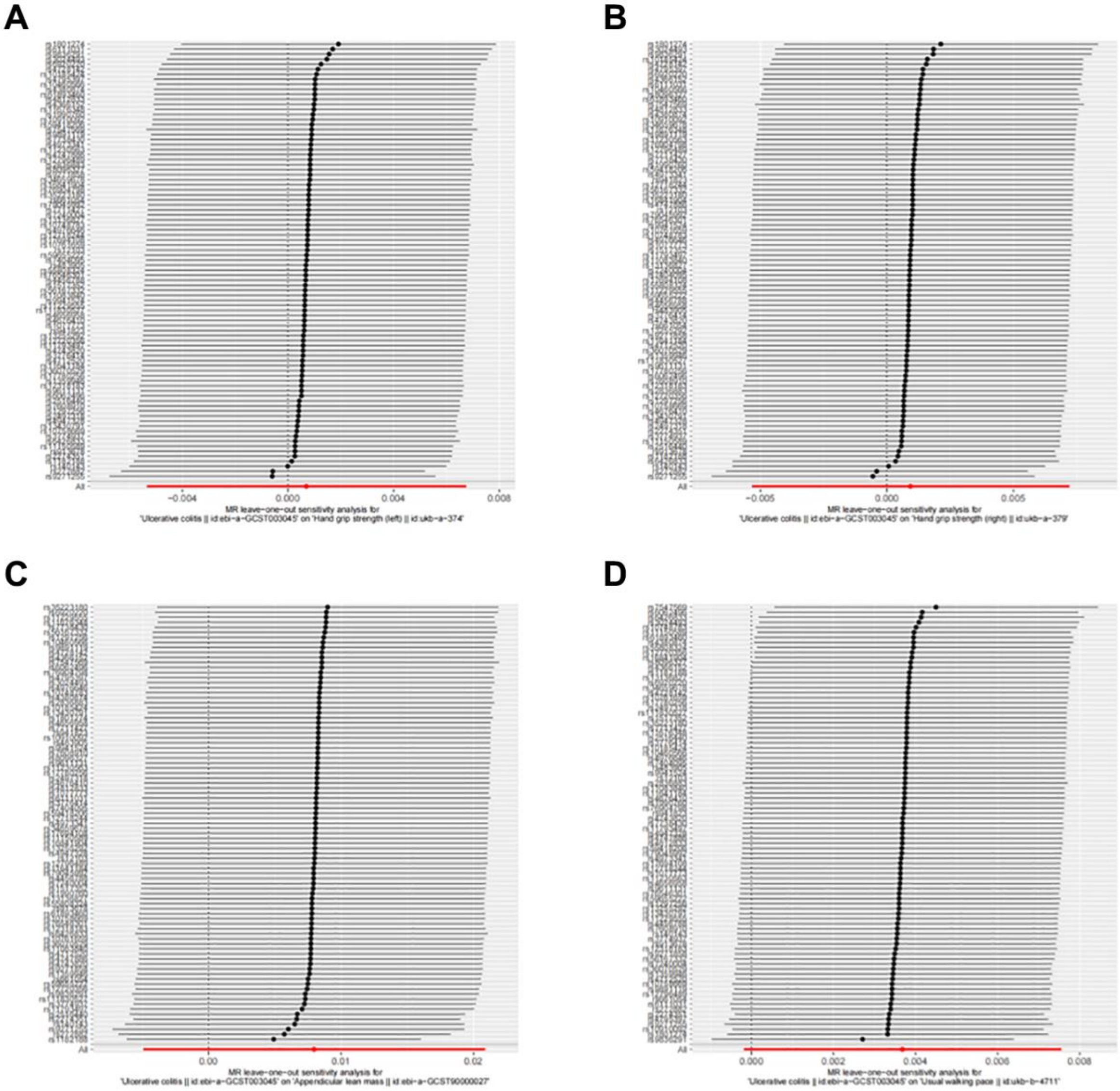

Supplementary Figure 3. Leave-one-out analysis in the MR analyses of the causal effect of UC on sarcopenia. (**A**) UC-grip strength (left). (**B**) UC-grip strength (right). (**C**) UC-ALM. (**D**) UC-Walking pace.

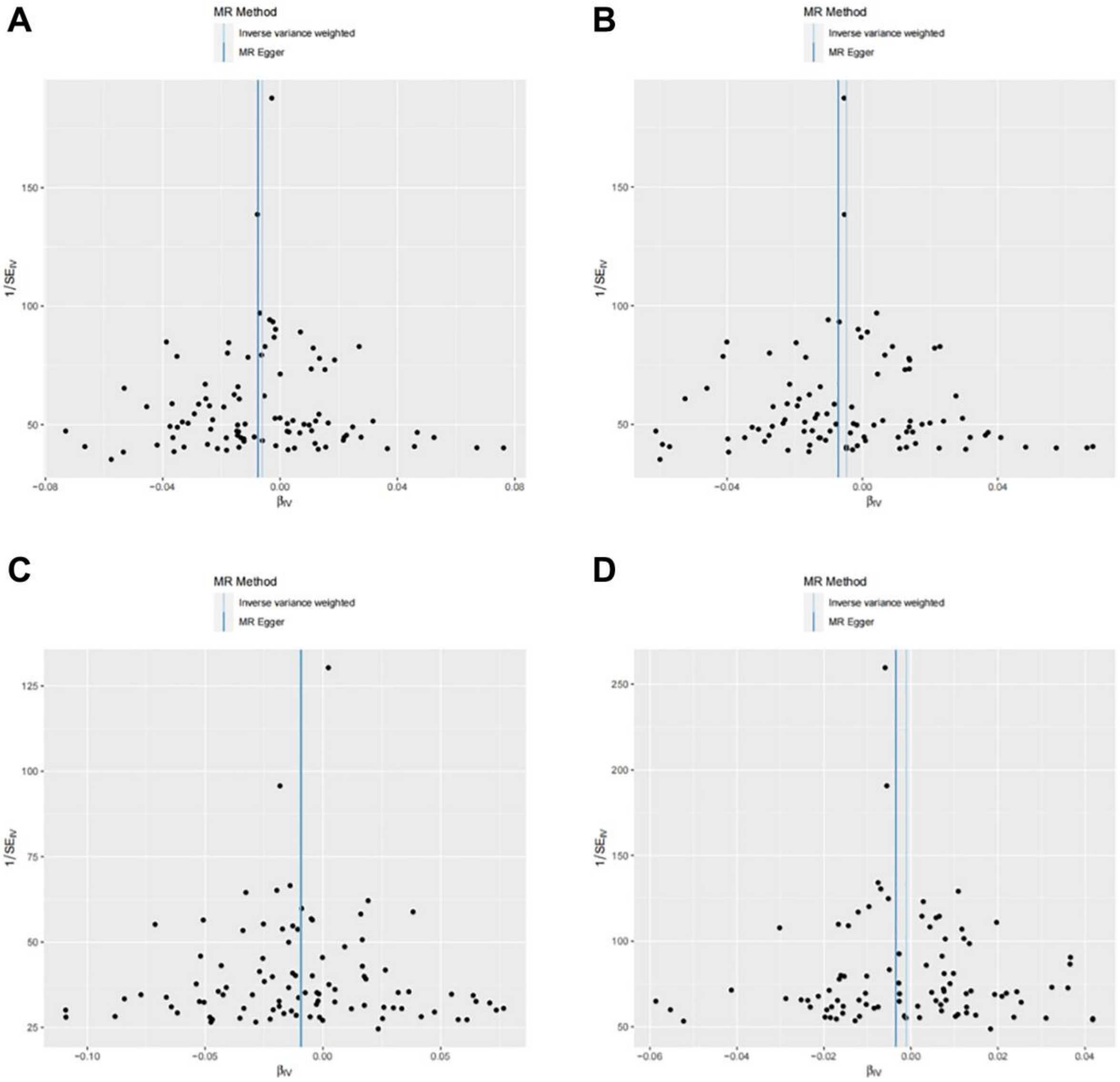

Supplementary Figure 4. Funnel plot for MR analyses of the causal effect of IBD on sarcopenia. (**A**) IBD-grip strength (left). (**B**) IBD-grip strength (right). (**C**) IBD-ALM. (**D**) IBD-Walking pace.

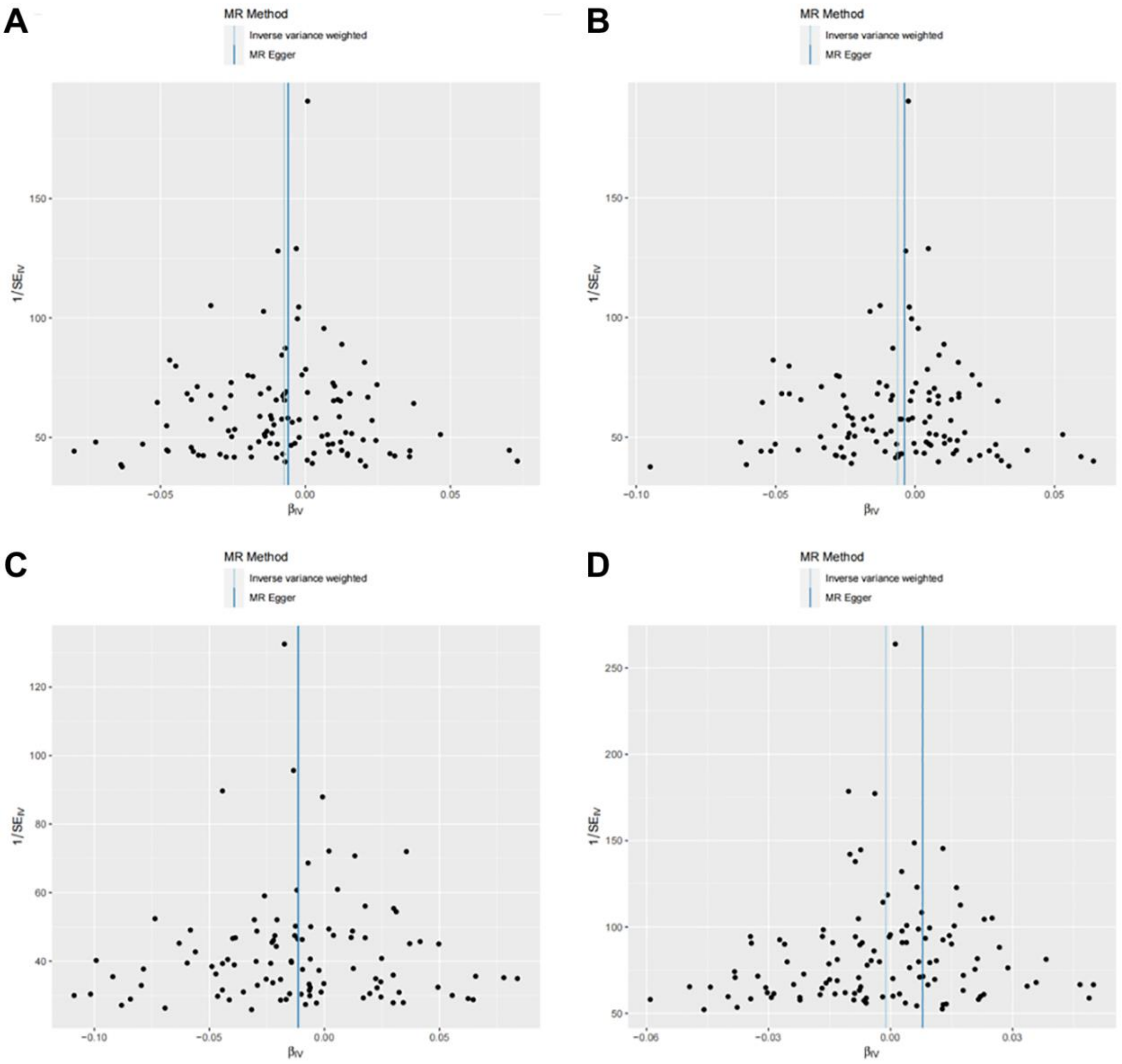

Supplementary Figure 5. Funnel plot for MR analyses of the causal effect of CD on sarcopenia. (**A**) CD-grip strength (left). (**B**) CD-grip strength (right). (**C**) CD-ALM. (**D**) CD-Walking pace.

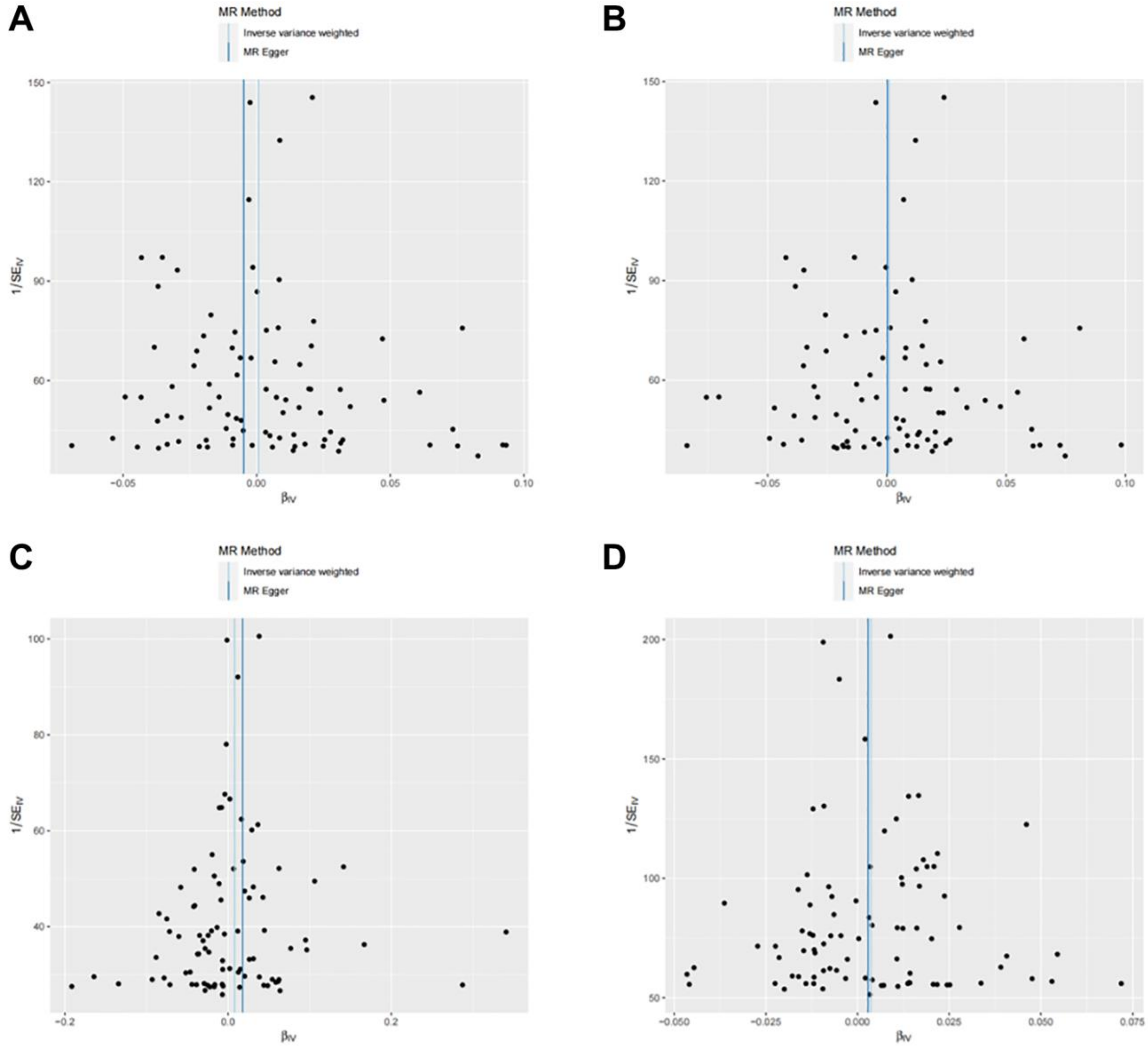

Supplementary Figure 6. Funnel plot for MR analyses of the causal effect of UC on sarcopenia. (**A**) UC-grip strength (left). (**B**) UC-grip strength (right). (**C**) UC-ALM. (**D**) UC-Walking pace.
